# Differential Effect of Aldosterone or Mineralocorticoid Receptor Overexpression on Retinal Inflammation

**DOI:** 10.1167/iovs.65.12.39

**Published:** 2024-10-25

**Authors:** Bastien Leclercq, Dan Mejlachowicz, Linxin Zhu, Laurent Jonet, Chadi Mehanna, Marianne Berdugo, Theano Irinopoulou, Fréderic Jaisser, Min Zhao, Francine Behar-Cohen

**Affiliations:** 1 Centre de Recherche des Cordeliers, Inserm UMRS1138, Université Paris Cité, Sorbonne Université, Paris, France; 2 Hôpital Américain de Paris, Neuilly-sur-Seine, Paris, France; 3 Ophthalmopole Cochin University Hospital, Assistance Publique-Hôpitaux de Paris, Paris, France; 4 INSERM UMR-S 1270, Institut du Fer à Moulin. Paris, France

**Keywords:** mineralocorticoid, corticoids, aldosterone, eye, retina, choroid, inflammation, transcriptomics, neuropathy

## Abstract

**Purpose:**

Overactivation of the mineralocorticoid receptor (MR) pathway is proinflammatory and contributes to the pathogenesis of diabetic retinopathy and of age-related macular degeneration. Excess of aldosterone, the specific MR ligand, is known to stimulate the production of proinflammatory cytokines and chemokines in extrarenal tissues and cells. In the RPE/choroid complex, aldosterone upregulated genes encoding proteins of the inflammatory response and downregulated genes encoding proteins involved in synaptic activity and neurotransmitters. Yet, cortisol, which is the main MR ligand in the eye, is a potent anti-inflammatory endogenous glucocorticoid. The aim of the present work was to better understand the role of MR activation in retinal inflammation either by acute injection of aldosterone or overexpression of the receptor.

**Methods:**

We first analyzed the retinal transcriptomic regulation induced by acute intraocular injection of aldosterone in the rat. Then, we used a transgenic rat overexpressing human MR (hMR) to also conduct retinal transcriptomic analysis as well as histological evaluation of the retina, retinal pigment epithelium and choroid.

**Results:**

Our results show that acute intravitreal injection of aldosterone is highly proinflammatory, upregulating pathways related to microglial activation, oxidative stress, cell death, and downregulating pathways related to glial/neuronal cells activity and proper neurotransmission. On the other hand, hMR overexpression mediates a low-grade inflammation in the retina, associated with notable choroidal inflammation and choroidal neuropathy.

**Conclusions:**

Consequences of hMR overexpression or aldosterone-injection on retinal transcriptome reveal very distinct pathological mechanisms, with only a few common genes regulated, most of them not being regulated in the same way. Although aldosterone is highly proinflammatory in the retina, MR overactivation in its physiologic milieu mediates a low-grade inflammation in the neural retina.

Upon activation by aldosterone, the mineralocorticoid receptor (MR), expressed in the kidneys exerts its physiologic role on water and electrolyte and on blood pressure control. In other tissues and organs, MR is rather activated by glucocorticoids (GCs) because cells do not express the hydroxysteroid dehydrogenase type II enzyme (HSD2), which by converting cortisol in the inactive cortisone, would allow aldosterone to reach MR. It has been recognized that MR pathway hyperactivation is pathogenic in cardiovascular, renal, and metabolic diseases, at least in part through proinflammatory immune effects and cytokines release.^1^^,^^2^ Aldosterone activates vascular and innate immune cells, such as dendritic cells, monocytes/macrophages, and adaptive immune cells.^3^^,^^4^ But macrophages can become proinflammatory upon MR activation, in the absence of aldosterone binding, as demonstrated in the heart using transgenic mouse models.^5^^,^^6^ In brain microglial cells, corticosterone exerts reverse effects upon binding to the glucocorticoid receptor (GR) or to the MR,^7^ and proinflammatory effects when activating MR.^8^ In addition, MR activation can be potentiated by oxidative stress independently from any ligands, through the Ras-related C3 botulinum toxin substrate 1 (Rac1).^9^ Altogether, these data indicate that both GCs and mineralocorticoids can induce proinflammatory effects depending on their dose, the GR/MR ratio and the cell types. In vivo environments in which both endogenous ligands are present, specific MR-mediated proinflammatory effects have been studied using either aldosterone excess or transgenic models of MR overexpression.

In the human and rodent retina, both GR and MR are expressed in microglia, glial cells, ganglion cells, inner retinal neurons, photoreceptors, and retinal pigment epithelial (RPE) cells, in retinal vascular endothelial cells, but also in choroidal vessels and in choroidal mast cells.^10^^–^^14^ As no aldosterone was detected in the retina or in the ocular media in rodents^15^ and in humans^16^ in the physiological state, MR is preferentially occupied by GCs, although aldosterone could still activate MR in retinal and choroidal vascular endothelial cells as well as in the RPE through its basolateral side.

In human RPE cells derived from pluripotent stem cells in vitro, that do not express HSD2, the specific activation of MR by either cortisol + GR antagonist or by aldosterone, resulted in differential transcriptomic regulations^17^ suggesting that different corticoids with different binding affinity for MR would result in different gene regulations. This information is of interest when selecting an optimal GC molecule for the treatment of retinal diseases in which retinal inflammation, including complement pathway and inflammasome activation, are pathogenic mechanisms. Glucocorticoids are commonly used to alleviate signs of inflammation, such as macular edema,^18^ but in wet age-related macular degeneration (AMD), intraocular GCs were ineffective^19^ and systemic exposure to GC can even be causative in central serous chorioretinopathy (CSCR).^20^ This paradoxical effect of GC could reflect MR/GR pathway imbalance,^11^ as described in the brain.^21^ In addition, in relevant animal models, in the human retina with diabetic retinopathy or with wet AMD,^13^^,^^22^ as well as in a model of retinopathy of prematurity,^23^ MR expression is increased and MR antagonism reduced retinal inflammation and activation of retinal microglial cells^24^ demonstrating the proinflammatory role of MR activation in the retina.

Because aldosterone is not detected in the vitreous in physiological conditions, this microenvironment is particularly adequate to specifically study MR activation by the addition of aldosterone, its natural ligand, in the vitreous. At low dose (20 nM in the vitreous), aldosterone induced retinal edema through the regulation of ion and water channels in glial cells^10^ and choroidal vasodilation through the regulation of the calcium depending potassium channel SK3.^11^ At a higher dose (100 nM in the vitreous), aldosterone caused posterior uveitis,^25^ upregulated genes in the RPE/choroid that encode proteins of the inflammatory response and downregulated genes encoding proteins involved in synaptic activity and neurotransmitters, involved in choroidal blood flow.^26^ Other groups showed that intravitreous aldosterone reduced the number of retinal ganglion cells (RGCs) in rats independently from ocular pressure.^27^ In addition, systemic aldosterone aggravated retinal pathology and inflammation in a mouse model of retinal vein occlusion,^28^ indicating that aldosterone could also aggravate retinal inflammation when the blood retinal barriers are disrupted. On the other hand, the chronic exposure to systemic aldosterone levels in the uninephrectomy-aldosterone-salt model in mice caused a choroidal phenotype close to the pachychoroid syndrome in humans, with enlarged choroidal vessels, migration of RPE cells, alteration of photoreceptor outer segments, and choroidal inflammation, but did not induce clinically detectable intraocular or retinal inflammation.^26^ Taken together, these experiments tend to show that MR activation by aldosterone excess is pathogenic in the retina and in the choroid, although the exact transcriptomic regulations induced by aldosterone in the neural retina have not been studied and which inflammatory pathways are activated are unknown.

Because MR is mostly occupied by GCs in the retina and MR is overexpressed in pathological conditions like diabetes or aging, but also that aldosterone and GCs may not regulate the same genes when they activate MR, transgenic models in which MR is overexpressed could reflect more accurately the effect of MR overactivation in the development of non-primarily inflammatory retinal diseases. We have previously described that mice overexpressing human MR (hMR) under the proximal P1 promoter^29^^,^^30^ develop a progressive pachychoroid epitheliopathy-like syndrome, that could result from a choroidal neuropathy.^31^ This animal model did not develop clinically detectable uveitis, although the transcriptional regulations induced by MR overexpression were not studied in the retina and histological signs of inflammation were not specifically analyzed.

In summary, MR pathway activation in the retina and in the choroid can be pathogenic and proinflammatory. But whether GCs could induce some form of retinal inflammation upon MR activation has not been evaluated and whether MR activation by GCs or by aldosterone results in similar transcriptomic regulations in the retina has not been explored.

To answer this question, we analyzed the transcriptional consequences of MR activation, either by acute intraocular injection of aldosterone or by hMR overexpression in a transgenic rat model with P1 promoter driven expression of hMR.

## Materials and Methods

### Intravitreal Aldosterone Injection in Rat Eyes and Transcriptomic Analysis

Experiments were approved by local ethical committees (#4488 and #2541, Charles Darwin). Eight-week-old male Lewis rats (*n* = 12) were kept in pathogen-free conditions with food and water ad libitum and housed in a 12-hour/12-hour light/dark cycle for 8 days before experiments were performed. The rats were anesthetized by intramuscular injection of ketamine (40 mg/kg) and Xylazine (4 mg/kg) and intravitreous injections were performed using microfine syringes and 31G needles under topical anesthesia (tetracaine 1%; Aldrich, Lyon, France). Six rats received 5 µL intravitreal injection of aldosterone diluted in 0.9% saline to obtain a concentration of 1 µM per injection, to expect a 100 nM final concentration in the vitreous. This concentration was chosen to reach 10 nM in the outer retina. Six control rats were injected with 5 µL saline. Both eyes of six rats per group were injected.

At 24 hours after the injections, the rats were euthanized by CO2 inhalation, the eyes were enucleated, and the neural retinas from the two eyes from the same animal were pooled for RNA-sequencing. Total RNA was extracted using a Precellys Homogenizer (Ozyme, Bertin) and a Qiagen RNeasy KIT (Cat. 74004, Qiagen, Hombrechitikon, Switzerland) and RNA was sequenced at the iGenSeq transcriptomic platform of the Brain and Spine Institute (Paris, France). The RNA quality was evaluated by capillary electrophoresis (Agilent 2100 Bioanalyzer system) and RNA with integrity numbers (RINs) ranging from 7.8 to 8.2 were accepted for library generation. The cDNA library of each sample was prepared with a KAPA mRNA Hyper Prep (Roche) for 75 bp paired-end reads, according to the manufacturer's instructions. Each of the cDNA libraries was indexed for multiplexing (2 × 60 million reads/sample), and indexed libraries were sequenced on one lane of the Illumina Nextseq 500 device. Data were recorded in the FASTQ format. For the differential gene expression (DGE) analysis, R version 4.4.0 and RStudio version 3.3.0 were used. After loading of the data and addition of the Rattus norvegicus annotations, data were filtered to select target sample and exclude the genes which are not expressed. Normalization and DGE analysis were performed using DESeq2 method (version 1.34; false discovery rate [FDR] fixed at 0.05 and log2 fold change (1/−1). Preranked Gene Set Enrichment Analysis (GSEA) was performed with Gene Ontology (GO) database terms, Kyoto Encyclopedia of Genes and Genomes (KEGG) pathways, and REACTOME pathways. Plot graphics were generated using ggplot2 library and the heatmap using pheatmap library.

### Transgenic Rat, hMR Transgene Validation

The transgene was built with the hMR under the proximal P1 promoter, as previously described in Le Menuet et al.^32^ Transgenic P1.hMR rats were generated by the Institut Clinique de la Souris (ILLKIRCH, France) on a Sprague-Dawley strain background (albinos model) and occasionally crossed with Long-Evans strain to obtain F1 pigmented animals for angiographic analysis. Overexpression of the transgene (ratio to endogenous rat MR) was validated in various ocular tissues by quantitative PCR (qPCR; Supplementary Fig. S1). P1.hMR and wildtype (WT) littermate (males and females) rats were bred in the Function Exploration Center (CEF; Campus des Cordeliers, Université Paris-Sorbonne, Paris) and kept in a 12:12 hour light dark cycle with drink/food ad libitum. They were kept in stabling until they reached 6 months old (for the RNA-sequencing study) or between 3 and 12 months old for histology studies. Experiments have been conducted in accordance with the European Communities Council Directive 86/609/EEC and the protocols have been approved by local ethical committees (#25158-2020041903191320 v3).

### Clinical Phenotyping of P1.hMR Rats

#### Retina Angiography and Spectral-Domain Optical Coherence Tomography

Indocyanine green angiography (ICG-A) was performed in 35 rats (17 WT and 18 P1.hMR). Nine rats were imaged at 16 months and the other ones between 5 and 6 months. There were 22 female rats (11 P1.hMR and 11 WT) and 13 male rats (7 P1.hMR and 6 WT). Rats were anesthetized using intraperitoneal (IP) injection of 100 mg/kg of ketamine (Clorkétam 1000, Virbac France) and 4 mg/kg of Xylazine (Rompun 2%, Bayer Healthcare, Loos, France). After pupil dilation, ICG (200 µL, 2.5 mg/mL INFRACYANINE, SERB, Paris, France) was injected intravenously in the tail of rats. Confocal ICG angiography was performed using Heidelberg Retina Angiograph II (Heidelberg Engineering, Inc., Dossenheim, Germany) to image choroidal and retinal vessels circulation and visualize ICG tissue staining. Posterior pole and peripheric images were recorded. Images were recorded between 1 and 3 minutes (early sequences) and between 10 and 15 minutes (late sequences) after ICG injection. Images were analyzed in a blind manner and graded according to the presence or absence of retinal vessel abnormalities, and abnormalities on choroidal vessels (tortuosity and dilation of choroidal veins, ICG leakage, and hyperfluorescent dots). The percentage of abnormalities was recorded in WT versus P1.hMR rats.

#### Electroretinograms

The visual function of P1.hMR animals (*n* = 6) and WT littermates (*n* = 8) was assessed by electroretinograms (ERGs) using a VisioSystem device by SIEM Bio-médicale (30900 Nimes, France). First, the animals were dark-adapted during 24 hours before being anesthetized using a mixture of Xylazine (10 mg/Kg) and Ketamine (100 mg/Kg) injected by an IP way. Then, one drop of atropine and tetracaine were applied to ensure pupil dilation and limit. Before putting on the electrodes, Ocrygel was applied to avoid drying of the eye surface. Scotopic ERG was performed in the dark with repeated light flashes (5 ms in duration) at increasing intensities, from 0.01 to 10 cd s/m2. For each intensity, the five flashes for responses were the average to measure a-wave and b-wave parameters. For photopic recordings, the animals were light-adapted for 5 minutes with a background light at 25 cd/m2, and then the response after a single blue, green, and white light flash of 10 cd s/m2 was recorded. At the end of the ERGs, the rats received a drop of artificial tears to avoid drying of the eyes. ERGs were analyzed using VisioSystem software to quantify a and b waves’ parameters (latencies and amplitudes). Statistical analyses were conducted in GraphPad Prism software using 2-way ANOVA followed by a Sidak’s multiple comparisons test (mean with SEM).

### Immunostaining Protocol

Rats were euthanized by IP injection of EUTHASOL VET (300 mg/kg, Dechra, Northwich, UK). After death, the eyes were quickly removed and fixed in 4% PFA for 2 hours. After fixation, the eyes were transferred in phosphate buffer saline (PBS) 1× and dissected to isolate the neuroretina and the eyes fundus (RPE, choroid, and sclera). Neuroretina were post-fixed by 10 minutes after incubation in frozen acetone before the immunostaining protocol. For immunostaining, rat sclera-choroid-RPE complexes and neuroretina were incubated for 1 hour in a pre-incubation solution (PBS 0.1M, 10% normal goat serum, 0.1% Triton X-100) at room temperature (RT). Then, the tissues were incubated either with a rabbit anti-IBA1 antibody (1:500, Ref:019-19741, Fujifilm Wako, Neuss, Germany), a monoclonal mouse anti-TUBB3 antibody (1:500, Ref: 801202, Biolegend, San Diego, CA, USA), or phalloidin-rhodamine (1:200, Thermo Fisher Scientific, France) in a buffer solution (PBS 0.1M, 5% normal donkey serum, 0.1% Triton X-100) during 4 days at 4°C. Following the first antibody incubation, the tissues were washed in PBS 0.1M 4 times during 10 minutes at RT. Afterward, incubations with the corresponding secondary antibodies (1:1000, Goat anti-rabbit and Goat anti-mouse antibodies Alexa Fluor 488/594 coupled) were performed for 3 hours at RT. The tissues were washed again for 4 × 10 minutes in PBS 0.1M before a quick DAPI staining (1:5000 in PBS 0.1M during 5 minutes). Finally, the tissues were flat mounted using Dako Omnis Fluorescence Mounting Medium (Agilent, Les Ulis, France) and images were acquired using a fluorescence microscope (model Olympus BX51, Olympus, Rungis, France).

### Retinal Microglia and Choroidal Nerve Quantification

To study the microglia morphology, 7 eyes of WT and 6 eyes of P1.hMR animals 6 months of age were studied. Nevertheless, quantification was not possible in 2 P1.hMR animals due to an important presence of IBA1+ macrophage-like shaped cells covering the microglia signals. For the quantification, several pictures were taken at 200 × magnification at different depths around the optic nerve and in the periphery of the retina for each animal. Using FIJI (Image J), Z-stacks were created in each zone to visualize the entire microglia anatomy. For each picture, approximately 60 microglia ramifications were tracked and measured using simple neurite tracer (SNT) in FIJI (Image J). Finally, variations in retinal microglia ramification length were compared between WT and P1.hMR animals using a nested *t*-test analysis (conducted in GraphPad Prism software). To quantify choroidal innervation, 6 flat-mounted choroids (3 WT littermates and 3 P1.hMR rats, all female rats) stained with TUBB3 were used. For each animal, 2 images were taken at 200 × magnification in the same zones both in the superior and inferior choroid (at the level of the inferior branch). Images were processed in Fiji (Image J) software to convert TUBB3 staining into greyscale, then greyscale levels were measured for each image to quantify TUBB3 signal, reflecting the choroidal innervation. Finally, greyscale levels were compared between WT and P1.hMR rats for superior and inferior choroids using a 2-way ANOVA analysis followed by a Sidak multiple comparison test (mean with SEM).

### Mast Cells Staining and Quantification

ICG incubation was previously used to visualize ICG transport and staining across the posterior segment.^33^ Sclera-choroid-RPE complexes of 5 P1.hMR and 3 WT littermates of 6 months of age were incubated at RT for 1 hour in a solution of 2% acetic acid, 30% ethanol, and 4% paraformaldehyde diluted in PBS 0.1M. Following incubation, the tissues were quickly washed 2 times for 1 minute in 95% ethanol before being stained for 5 minutes in 0.25% toluidine blue. Then, the tissues were washed 2 times in 95% ethanol, dehydrated, and mounted on a slide using Eukitt mounting medium (VWR Avantor, Rosny-sous-Bois, France). This protocol has already been validated by our team and allows to only stain mast cells (Bousquet et al. 2015). For quantification, multiple pictures were taken using a photonic microscope (Olympus BX51, Olympus, Rungis, France) at 40 × magnification to cover the whole choroid. Then, the mast cells were counted using the FIJI (Image J) cell counter in the superior (above long posterior ciliary arteries [LPCAs]) and inferior (below LPCA) zones of the choroid. Statistical analyses were conducted in GraphPad Prism. The total number of counted choroidal mast cells were compared between the two genotypes using an unpaired *t*-test analysis. To compare the mast cells’ repartition between the superior/inferior choroid depending on the genotype, 2-way ANOVA analysis with Sidak multiple comparison analysis were performed (mean with SEM).

### ICG Incubation, Staining, and Image Acquisition

Rats (9 WT and 9 P1.hMR) aged between 3 and 12 months old were euthanized by IP injection of EUTHASOL VET (300 mg/kg, Dechra, Northwich, UK). After enucleation and dissection of the anterior segment, the posterior segment of the eyeball including the retina, choroid, and sclera complexes were incubated immediately for 45 minutes in DMEM medium (41965039, Thermo Fisher Scientific, Illkirch-graffenstaden, France) with 1% fetal bovine serum (10270106, Thermo Fisher Scientific) and 10% ICG (2.5 mg/mL, Infracyanine) at 37°C (5% CO2). After washing with DPBS, the neuroretina was removed. The RPE–choroid–sclera complexes were fixed with 4% paraformaldehyde, and then post-fixed with acetone for 10 minutes at −20°C. RPE-choroid-sclera complexes were flat mounted with Dako Omnis Fluorescence Mounting Medium (Agilent, Les Ulis, France) and counter stained with DAPI (1:5000) for nuclei staining.

A confocal microscope (STELLARIS 5, Leica Microsystems, Wetzlar, Germany) and far-red laser excitation (685 nm with a White Light Laser) were used to visualize and acquire ICG signals.

RPE-choroid-sclera complexes were also used for IBA1 immunostaining and phalloidin staining (4 WT and 4 transgenics) following the immunostaining protocol described in the “Immunostaining protocol” section. To acquire images, the same confocal was used.

### Semithin and Transmission Electron Microscopy Preparations

Three 1-year-old male P1.hMR rats and their 3 WT littermates were euthanized by IP injection of EUTHASOL VET (300 mg/kg, Dechra, Northwich, UK) and the eyes were fixed with 2.5% glutaraldehyde (2 hours) for semithin/ultrathin transmission electron microscopy (TEM) sections. The eyes were washed in cacodylate buffer for 2 hours and post-fixed by osmium tetroxide (OsO4 2% in cacodylate buffer). Then, the samples were dehydrated in a graded alcohol series (70%, 90%, and 100%), oriented, and embedded using LX112 embedding kit (Ladd Research Industries, Williston, VT, USA). The polymerization was performed at 60°C for 2 consecutive days. Blocks were finally cut using an ultramicrotome, either for semithin sections (1 µm) stained with 1% toluidine blue or ultrathin sections (80 nm). Semithin sections were observed, and images were acquired by light microscopy (Olympus BX51, Rungis, France) under immersion objectives. Ultrathin sections were contrasted and observed by TEM at Jacques Monod Institute (Institut Jacques Monod, CNRS - Université Paris Cité, Paris).

### Tissue Preparation and Transcriptomics Analysis of P1.hMR Neuroretinas

Six months old female P1.hMR rats (*n* = 5) and their age and sex-matched WT littermates (*n* = 2) were euthanized by IP injection of EUTHASOL VET (300 mg/kg, Dechra, Northwich, UK) and the eyes were removed and quickly dissected on ice. Neuroretinas were isolated and frozen in liquid nitrogen. RNA-extraction was performed within a few days using Qiagen RNeasy KIT (Cat. 74004, Qiagen, Hombrechitikon, Switzerland) and 500 ng of extracted RNA were sent for RNA sequencing, as described above at the iGenSeq transcriptomic platform of the Brain and Spine Institute (ICM, Paris, France). The quality of raw data was evaluated with FastQC. Poor quality sequences and adapters were trimmed or removed with fastp, using default parameters, to retain only good quality paired reads. Illumina DRAGEN bio-IT Plateform (version 3.8.4) was used for mapping on rn7 reference genome. Library orientation, library composition, and coverage along transcripts were checked with Picard tools. The transcriptomic data were analyzed on the online platform of the Paris Brain institute (quby.icm-institute.org). First, differential expression analysis using DESeq2 method was assessed to determine the DEGs (adjusted *P* value [pFDR], FDR threshold set at 0.05, and log2 fold-change threshold set at 0). Then, an enrichment analysis was conducted (under over-representation analysis) in different gene-set collections (MSigDB database) including Hallmark gene sets, Reactome gene sets (Reactome subset of Canonical pathways), and GO gene sets.

## Results

### Acute Intraocular Aldosterone Upregulates Genes of Inflammatory, Oxidative Stress, and Metabolism in the Neural Retina

**Figure 1.**
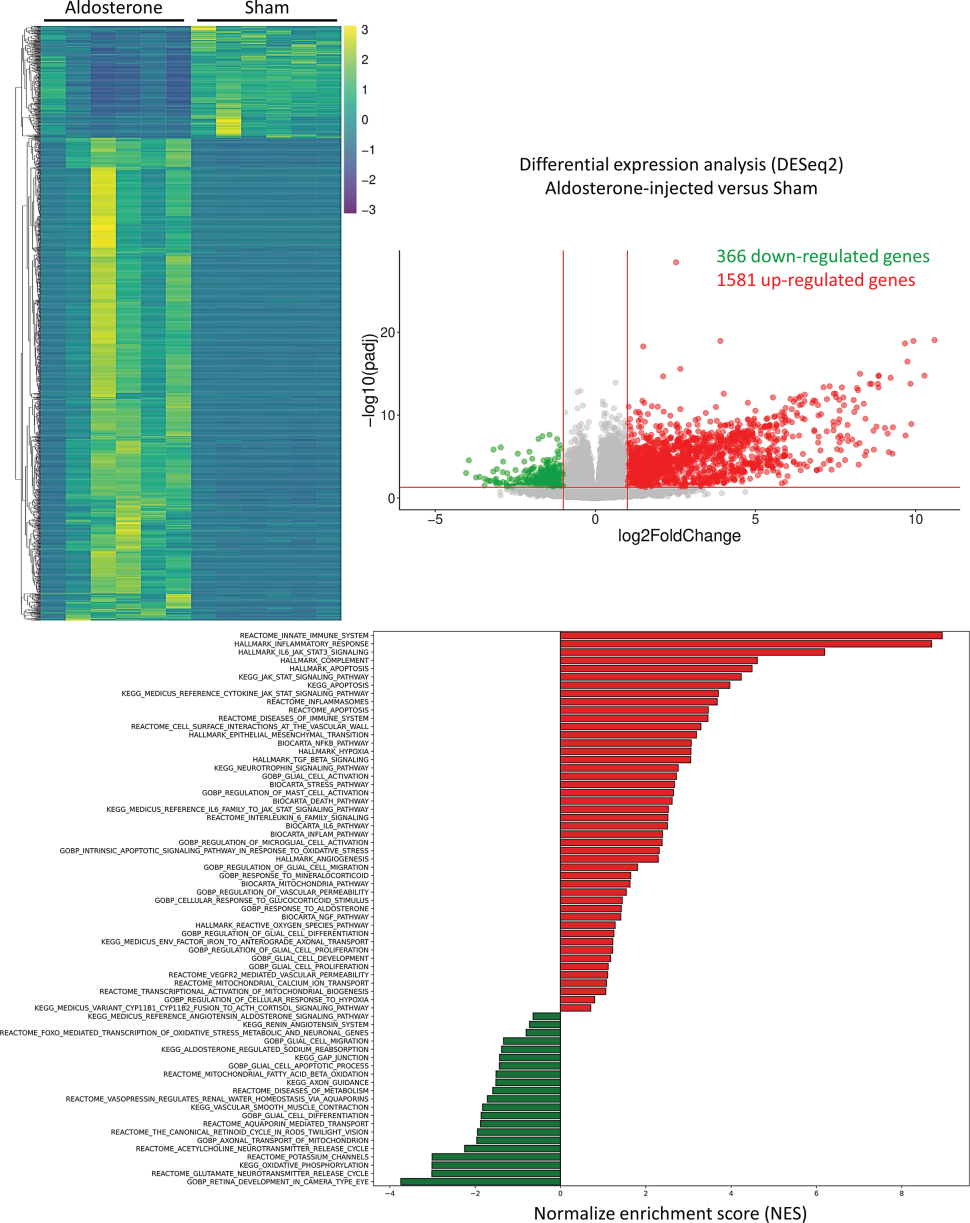
**Transcriptomics analysis of rat neural retina 24** **hours** **after acute intravitreal aldosterone injection.** Rats either received acute intravitreous aldosterone injection (the aldosterone group *n* = 6) or saline solution (the sham group *n* = 6). After 24 hours, neural retinas were extracted for RNA-sequencing analysis. Differential analysis by DESeq2 method results in 366 downregulated genes and 1581 upregulated genes in the aldosterone group compared to the sham animals (see the Heatmap on the *left* and Volcano on the *right*). Pathway’s analyses (bar-plot above) show that aldosterone injection increase inflammation through IL-6-Jak-Stat3, microglia activation, oxidative stress, and apoptosis processes (positive NES) while decreasing glutamate/acetylcholine neurotransmission, vascular smooth muscle cell contraction, aquaporin-mediated transport, mitochondrial fatty acid beta oxidation, and axonal transport of mitochondrion (negative NES).

Eight week old rats received either intravitreal aldosterone injection (aldosterone group) or saline injection (sham control group) that causes a clinically detectable retinal inflammation with infiltrating cells in the retina and in the vitreous.^14^^,^^26^ At 24 hours, 1947 genes were significantly regulated by aldosterone in the neural retina, with 366 downregulated and 1581 upregulated (Fig. 1). Enrichment analysis (GO, KEGG, KEGG MEDICUS, BIOCARTA, Reactome, and Hallmark) were performed to highlight regulated pathways (see Fig. 1, bar-plot). Response to mineralocorticoid (GOBP), response to GR stimulus (GOBP) and response to aldosterone (GOBP) are found positively regulated (in red), with several other pathways related to inflammation through IL6-Jak-Stat3, microglia activation, oxidative stress, and apoptosis processes. Among negatively regulated pathways (in green), a large part is related to glutamate/acetylcholine neurotransmission, vascular smooth muscle cell contraction, aquaporin-mediated transport, mitochondrial fatty acid beta oxidation, and axonal transport of mitochondrion. A complete list of DEGs is available in Supplementary File S1. Among the regulated genes, a number of them are known to contribute to microglia/ macrophage polarization, such as *Pxrd3*, *Tlr4/MYD88*, *Notch-Arg1*, *Atf3*, *Tlr2* and *4*, *Wnt4*, *Tspo*, *Stat3*, *Sall1*, *Pentraxin3*, *Periplin2*, aldose reductase, and *S100A9.* This list of genes represents specific MR molecular targets in the neural retina and it will be used to compare the transcriptional regulations induced by cortisol that activates both MR and GR with similar affinity.

Because MR overactivation is a pathogenic mechanism, that MR is overexpressed in several retinal diseases and can be activated by other ligands or mechanisms than aldosterone (that could induce differential regulations), we generated a transgenic rat model overexpressing the human MR under the control of P1 promoter (P1.hMR rats). This model is now used to analyze the consequences of MR overactivation by its endogenous ligands in the different eye compartments.

### P1.hMR Rats Present Choroidal Vascular Changes but no Clinical Signs of Retinal Inflammation

#### Choroidal Vascular Abnormalities

**Figure 2.**
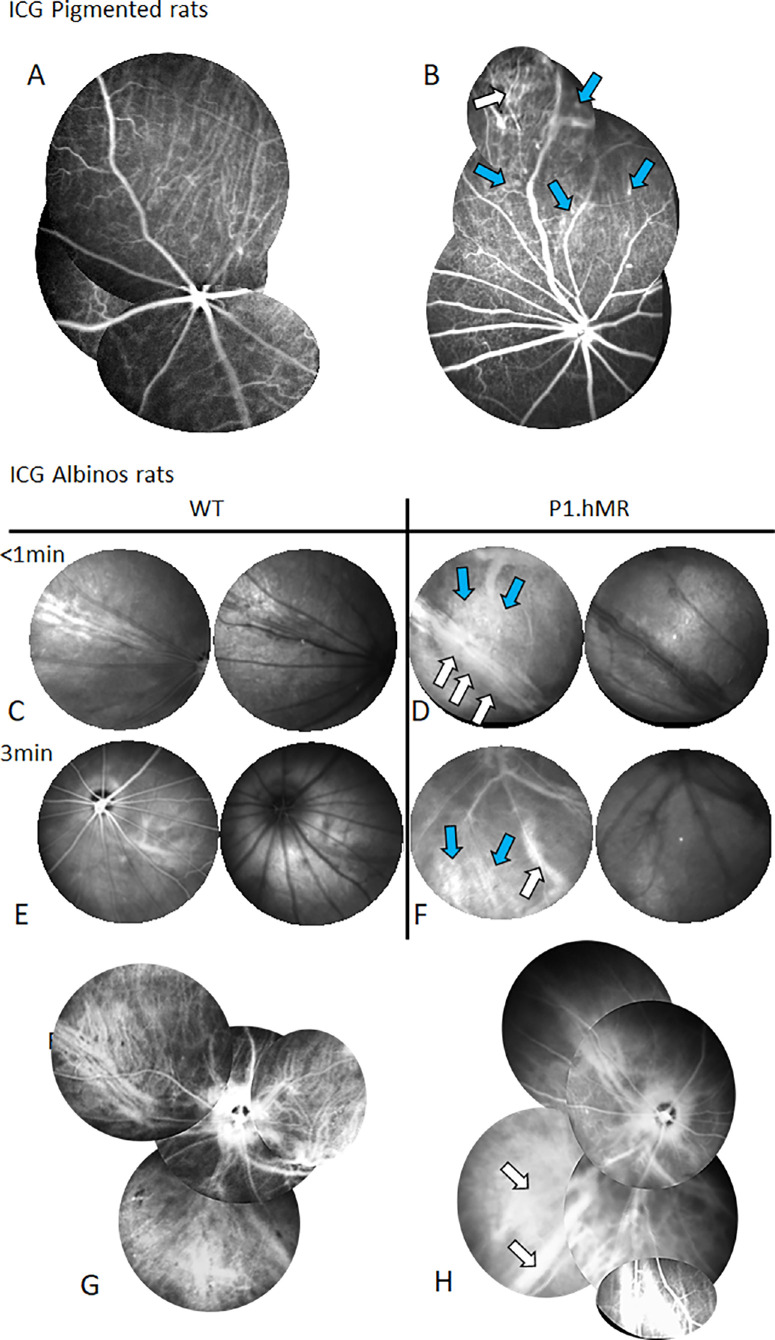
**ICG angiography in P1.hMR rats reveals choroidal vascular abnormalities**. ICG-A in pigmented rats (**A** and **B** between 2 and 3 minutes) and in albino rats (**C**, **D** and **E**, **F**). Normally perfused retinal and choroidal vessels are seen in WT rats **A**, but in P1.hMR rats, dilated choroidal veins (*white arrow*) and micro aneurysm together with hyperfluorescent spots (*blue arrows*) can be observed **B**. In Albino rats, the early ICG-A sequence allows to visualize the long ciliary vessels and nerves in WT rats **C** and to recognize the dilated and leaky choroidal vein **D** (*white arrows*) in the transgenic animal, the dilated vorticous veins and hyperfluorescent plaque **D** (*blue arrows*). Images taken at the posterior pole and at a later time (3 minutes) show dilated choroidal veins **F** (*white arrow*) and focal area of hyperfluorescence **F** (*blue arrow*) in the transgenic rat only. (**G**, **H**) Angiography pictures editing for better visualization in WT **G** and P1.hMR animals **H**. Important veinous vasodilation and leakages can be observed within the choroid in P1.hMR rats (*white arrows*) in **H** compared to WT littermates in **G**.

In WT rats, no abnormalities or retinal vessels were observed, and dilations of choroidal veins were observed in 4 of 17 animals (23%), but no leakage was observed in any of the rats. In P1.hMR rats, choroidal vascular abnormalities were observed in 15 of 18 rats (83%) and vascular leakage, aneurysmal dilations, or hyperfluorescent ICG dots were observed in 9 of 18 transgenic rats (50%; 6/8 female rats and 3/6 male rats). In albino rats, choroidal vessels can be visualized more easily and intense ICG leakage is observed around the long posterior ciliary vein that is surrounded by large choroidal nerves (Fig. 2D).

#### ERGs Analysis of P1.hMR

**Figure 3.**
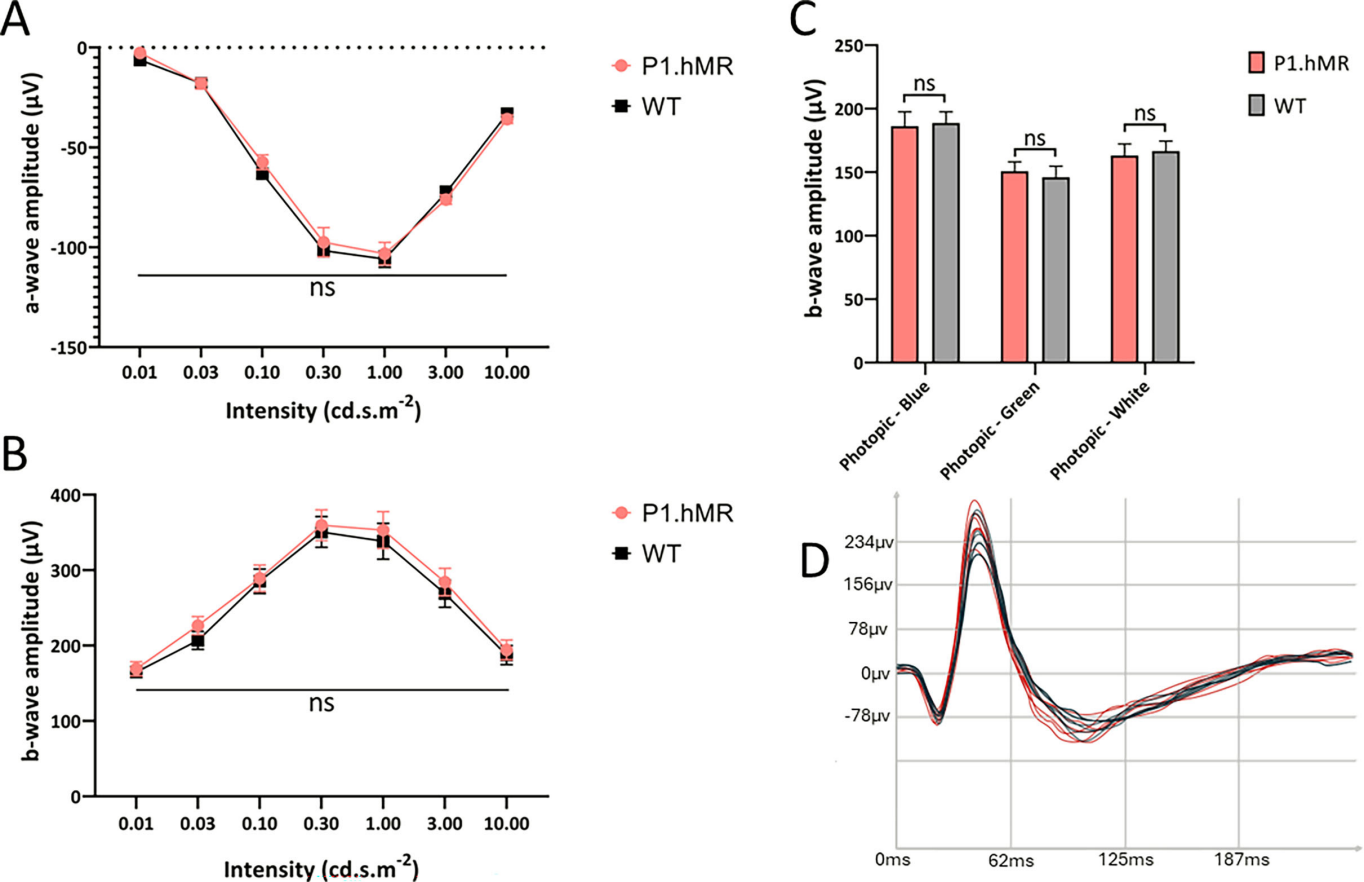
**P1.hMR animals show no alteration in electroretinogram.** Scotopic a-wave (**A**) and b-wave (**B**) amplitudes show similar variations between WT and P1.hMR animals. In photopic conditions (**C**), no significant difference was seen in the b-wave amplitude between the two genotypes, both under blue, green, and white light stimulation. Whereas the ERG is normal, P1.hMR animals show no signs of visual alteration. (**D**) P1.hMR (in *red*) and WT littermates (*black*) scotopic ERG responses following 3 cd.s.m^−2^ light pulse intensity.

To evaluate the potential consequences of hMR overexpression on electrophysiological visual responses, ERG recordings were performed in P1.hMR and WT littermates (female and male rats) 6 months old. Scotopic and photopic responses were analyzed (Fig. 3). Overall, there are no significant alterations in scotopic a-wave or b-wave amplitudes between WT and P1.hMR animals, with similar variation both in female and male rats (not differentiated; see Figs. 3A, 3B). Similarly, in photopic conditions under blue, green, and white light stimulation, there are no significant differences in the b-wave amplitude between the two genotypes (Fig. 3C). These results indicate that young P1.hMR animals do not exhibit any electrophysiological signs of visual dysfunction. On anatomic sections, no retinal abnormalities were observed except a photoreceptor segment’s elongation.

### P1.hMR Rats Show Signs of Microglia and Choroidal Mast Cell Activation and of Low-Grade Inflammation in the Neural Retina

#### Morphological Changes of Choroidal IBA1+ Cells and Retinal Microglia

**Figure 4.**
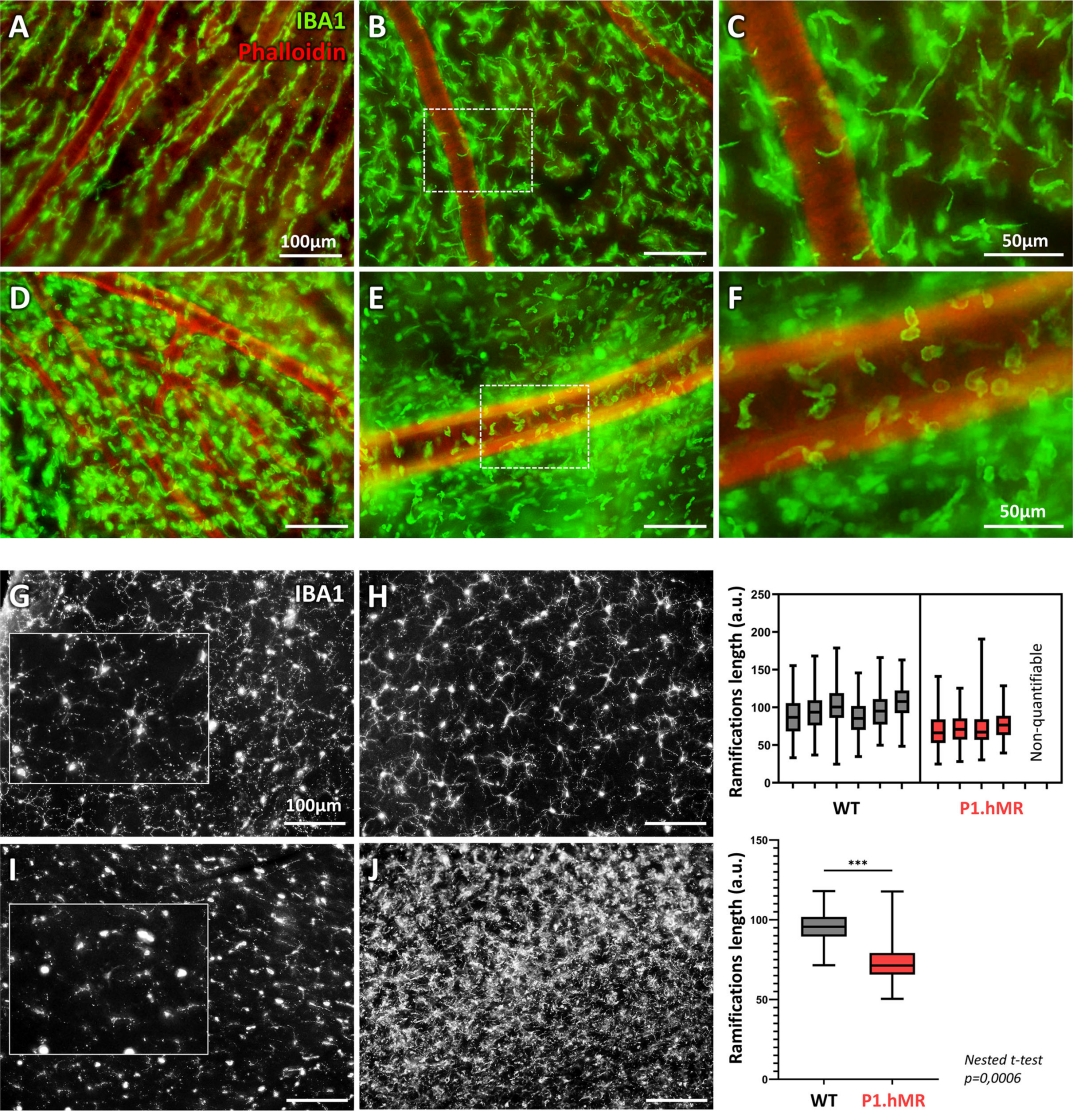
**Morphological changes of IBA1+ choroidal cells and retinal microglia activated morphology in P1.hMR.** Actin staining by phalloidin-rhodamine (*red*) and IBA1 immunostaining (*green*). (**A**–**C**) In WT rats, choroidal IBA1+ cells are ramified and homogenously distributed within the choroid, notably around the arteries. (**D**–**F**) In P1.hMR transgenic rats, IBA1+ cells shift toward a “round’ shape morphology while losing their organization around large choroidal vessels. The changes in the morphology of IBA1+ could indicate a proinflammatory state in the choroid of P1.hMR rats. Retinal microglia stained by IBA1 immunostaining show a well ramified morphology in WT animals (**G**, **H**), whereas the ramifications are significantly decreased in P1.hMR animals (**I**, **J**; graphics). In two P1.hMR animals, ramifications were not measurable due to an important presence of IBA1+ macrophages-like shaped cells **J**. The reduced microglia ramification and potential macrophages infiltration in P1.hMR animals could indicate a proinflammatory state in the neuroretina under hMR overexpression.

Immunostaining for IBA1 and actin revealed distinct morphological alterations in choroidal IBA1+ cells between WT and P1.hMR rats (Fig. 4). In WT rats, IBA1+ cells exhibit a ramified morphology and are evenly distributed throughout the choroid, with a pattern organization around the arteries (see Figs. 4A–C). However, in P1.hMR transgenic rats, a significant shift in morphology is observed, with IBA1+ cells adopting a round shape and displaying a loss of organization around large choroidal vessels (see Figs. 4D–F). This transition in morphology suggests a potential proinflammatory state within the choroid under hMR overexpressing conditions. In retinal tissues, notable differences in the morphology of microglia between WT and transgenic animals are also noticeable. Whereas retinal microglia display a well-ramified morphology in WT animals (see Figs. 4G, 4H), P1.hMR retinal microglia exhibit a significant reduction in microglial ramifications, indicating a shift toward the activated state (see Figs. 4I, 4J). Moreover, in some P1.hMR animals, the presence of macrophage-like shaped cells further suggests potential infiltration of inflammatory cells into the neuroretina (see Fig. 4J).

### Mast Cell Numbers and Distribution in the Choroid

**Figure 5.**
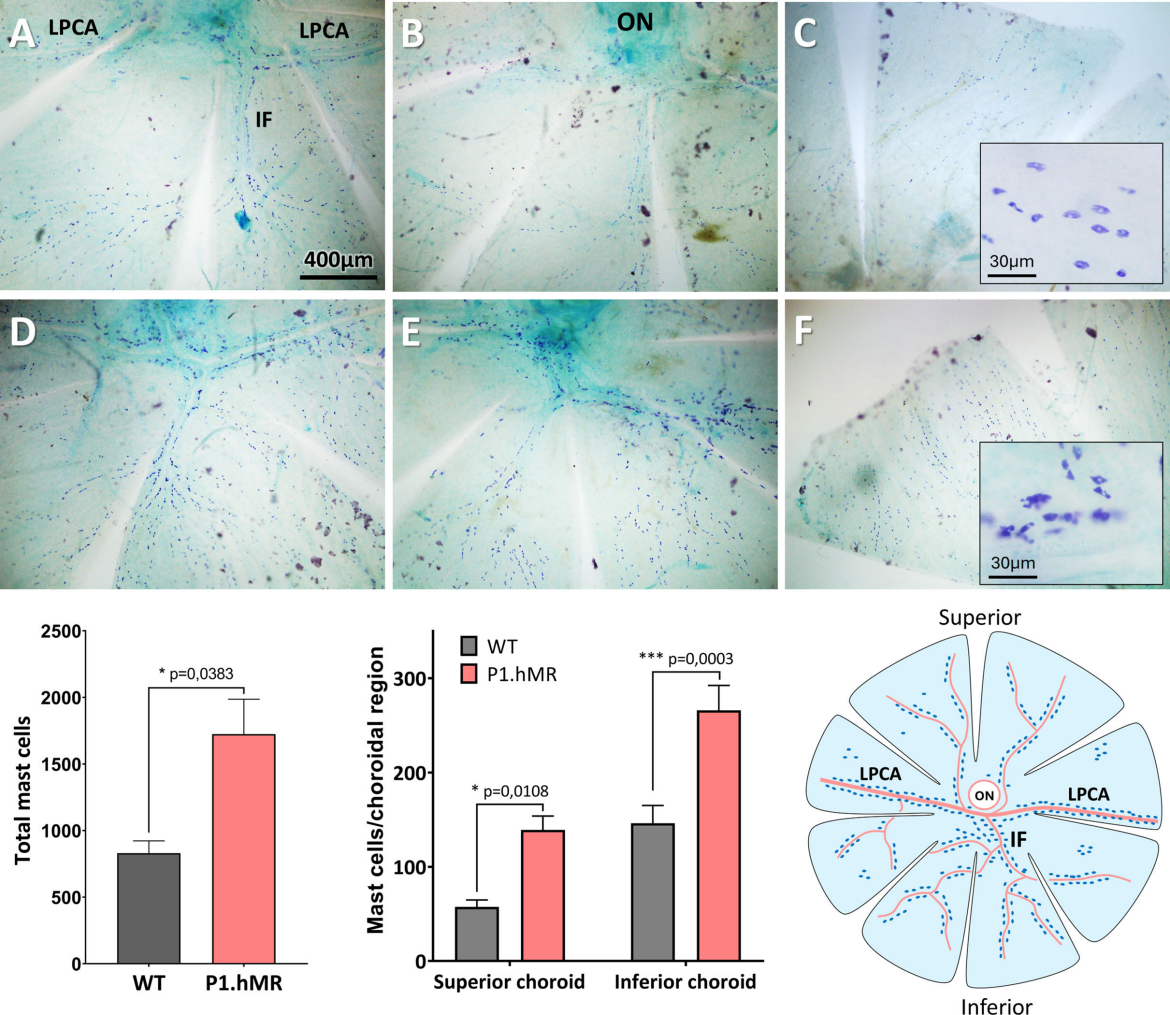
**P1.hMR animals display increase mast cells in the choroid.** Toluidine blue staining on whole-mounted choroid allows to visualize choroidal mast cells population. (**A**–**C**) choroidal mast cell repartition along the long posterior ciliary arteries (LPCAs) and the inferior branch (IF) **A** and **B** and the superior choroid **C** in WT littermate animals. (**D**–**F**) Repartition of choroidal mast cells in P1.hMR animals. (*inserts* **C**–**F**) High magnification of choroidal mast cells organized around the choroidal vasculature. Mast cells in P1.hMR **F** are more often darker and aggregated compared to WT animals **C**. Total mast cell counting reveals a significant increase of this cell population in P1.hMR animals (unpaired *t*-test). This increase is significant both in superior and inferior choroid compared to WT animals (2-way ANOVA and Sidak multiple comparison). Increase of mast cells population in P1.hMR could indicate a proinflammatory environment in the choroid and an amplified response to stress.

We previously showed that choroidal mast cells express MR^14^ and that choroidal neuropathy is induced in P1.hMR rats.^31^ Because mast cells are under neural control, it was interesting to study the consequence of MR overactivation on choroidal mast cells in the albino P1.hMR rat. Toluidine blue staining and counting of whole-mounted choroid reveal a notable increase in the number of stained mast cells in P1.hMR animals compared to WT littermates (Fig. 5). This increase is noticeable along the long posterior ciliary arteries (LPCAs), inferior branch (IF), and superior choroid (see Figs. 5D–F), but also in the intervascular spaces. Statistical analysis confirms a significant increase in mast cell population in both superior and inferior choroid regions of P1.hMR animals compared to WT littermates.

**Figure 6.**
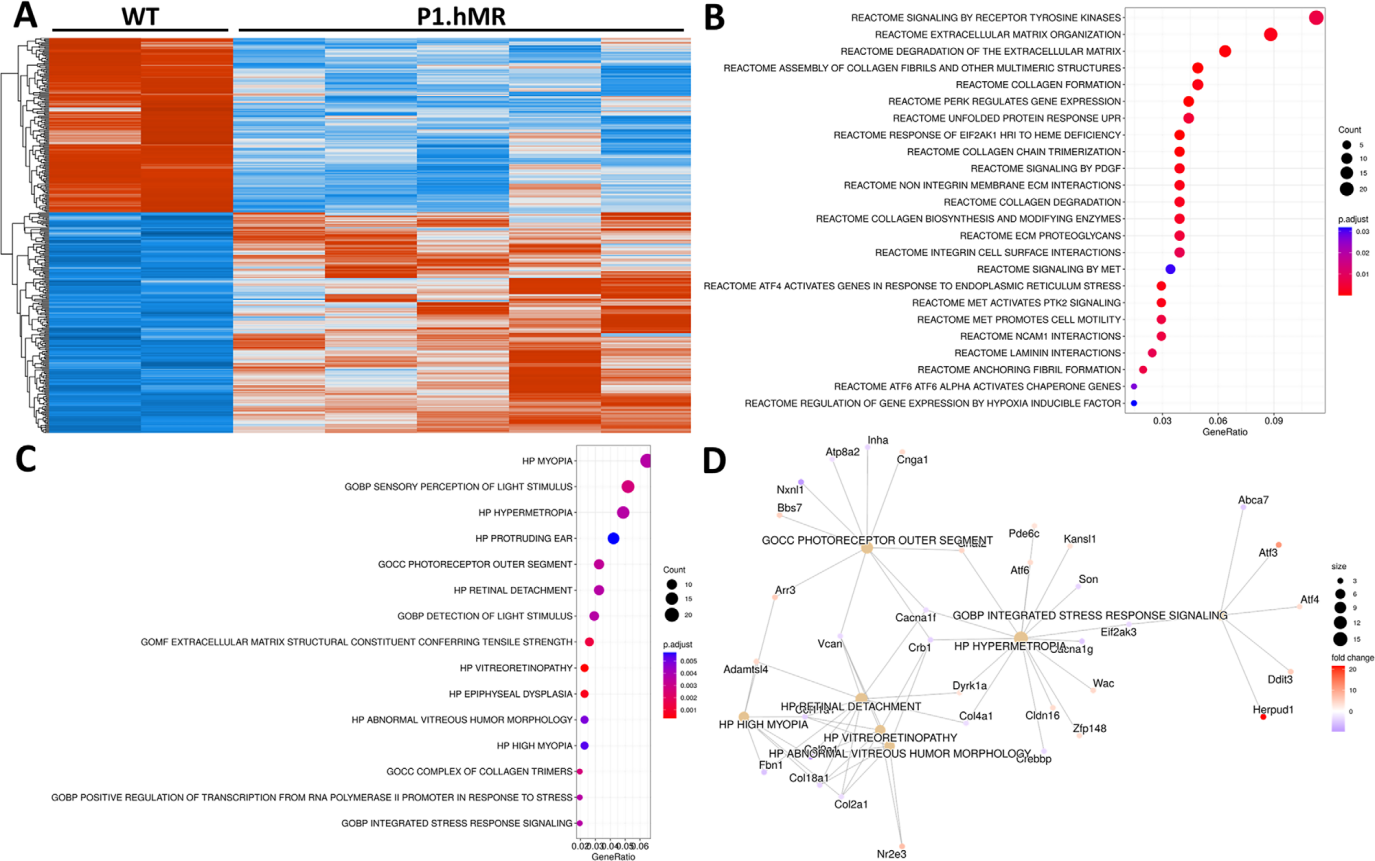
**Transcriptomic analysis of P1.hMR rats’** **neural retina.** (**A**) Heatmap displaying differentially expressed genes in P1.hMR rats (*n* = 6) compared to WT littermates (*n* = 2). One hundred fifty-two genes are downregulated (in *blue*) whereas 192 genes are upregulated (in *red*). (**B**) Dot plot of Reactome genes set significantly over-represented in P1.hMR animals. Dot plot (**C**) and gene-concept network (**D**) of significantly over-represented GO gene set.

To characterize the retinal transcriptome under hMR overexpression, neural retinas from P1.hMR animals and WT littermates were studied by RNA sequencing (Fig. 6). A total of 344 genes were found to be significantly regulated, with 152 downregulated and 192 upregulated in the neural retina. Regulated genes were analyzed within the GO and Reactome databases, showing multiple regulated pathways related to the response to hypoxia unfolded protein response stress and to extracellular matrix organization. Interestingly, human phenotype (HP) of hypermetropia and myopia and retinal detachment were identified. Raw data are available on Gene Expression Omnibus (GSE266379), and a complete list of differentially expressed genes is available in Supplementary File S2. Globally, genes regulated in the retina of P1.hMR rats encode proteins that have function on the RPE/ choroid physiopathology, such as homocysteine inducible ER protein with ubiquitin like domain 1 (Herpud1), activating transcription factor 3 (ATF3), and the endothelial Pas domain protein 1 (Epas1) which encodes a paralog of HIF-1a, Htra1, and high-temperature requirement A1 that encodes a secreted serine protease, and VEGF. Genes encoding proteins that intervene in the RPE/ neural retina interface are also regulated in the P1.hMR neural retina, such as the chloride intracellular channel 4 (CLIC4) and the retbindin protein. Gene encoding proinflammatory proteins were counterbalanced by genes involved in the regulation of the inflammatory response, corresponding to the observed mild and infra clinic retinal inflammation.

### Signs of RPE/Choroidal Inflammation and Pathology

**Figure 7.**
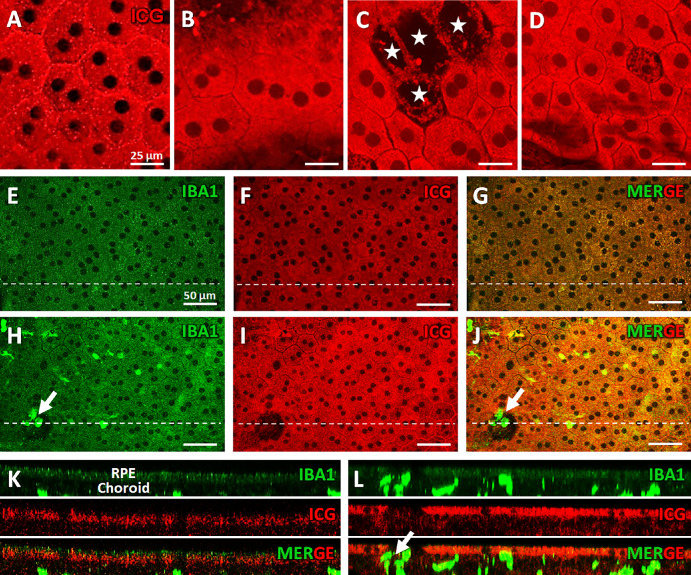
**RPE structural alteration and choroidal IBA1+ cells proximity in P1.hMR rats.** ICG signals in the RPE of WT (**A**, **F**) or P1.hMR (**B**, **C**, **D**, **I**) rats. P1.hMR animals show several zones of RPE cells loss **C** and **D** and an inhomogeneous ICG signal (after ICG incubation) within RPE cells **B** and **D** compared to the more “vesicular” signal observed in WT rats **A**. IBA1 immunostaining combined with ICG signal in P1.hMR animals (**H**–**J**) compared to WT (**E**–**G**). These pictures are visualized in Z-stack along the *dotted white line* in WT (**K**) and P1.hMR animals (**L**). IBA1/ICG staining show that IBA+ positive cells are juxtaposed to the RPE in P1.hMR rats and seem go through the RPE where cells are missing (*white arrow* in **H** and **J**). P1.hMR exhibits RPE structural changes, including specific pattern in ICG loading, pigment redistribution/loss and RPE cells associated with recruitment of IBA1+ choroidal cells.

Analysis of RPE structural alterations in P1.hMR rats reveals several abnormalities compared to WT animals. ICG is used to analyze the macromolecular transports in the RPE and help interpret the ICG-A imaging.^33^ RPE cells in WT rats have accumulated ICG inside vesicles, located in the cytoplasm and at the membrane because ICG is transported at least in part through caveola-mediated transport (Fig. 7A). In P1.hMR rats, structural abnormalities of the RPE organization can be better visualized on flat-mounted RPE/ choroid from albino rats (see Figs. 7H–J), showing zones of RPE cell loss (Figs. 7C, 7D) and major polydispersity (see Figs. 7B, 7C, 7D, 7I). ICG is inhomogenously distributed in RPE cells (see Figs. 7B, 7C, 7D, 7I) with a loss of vesicular location indicating alteration of macromolecular transports across the RPE barrier. Furthermore, IBA1 immunostaining combined with ICG signal demonstrate a proximity of IBA1+ choroidal cells to the basal side of RPE, suggesting potential interaction between these cell types (see Figs. 7E–L). The presence of IBA1+ cells is also noticeable in zones where RPE cells are lost and where IBA1 positive cells are passing through the RPE barrier toward the outer retina (see Figs. 7H, 7J, 7L). The xz three-dimension (3D) images show the location of IBA1 positive cells at the contact of the basolateral side of the RPE and passing across the barrier (see Fig. 7L). These findings highlight pathological RPE structural changes associated with recruitment of choroidal IBA1+ cells in P1.hMR rats, indicating a complex interplay between these components and inflammatory processes.

**Figure 8.**
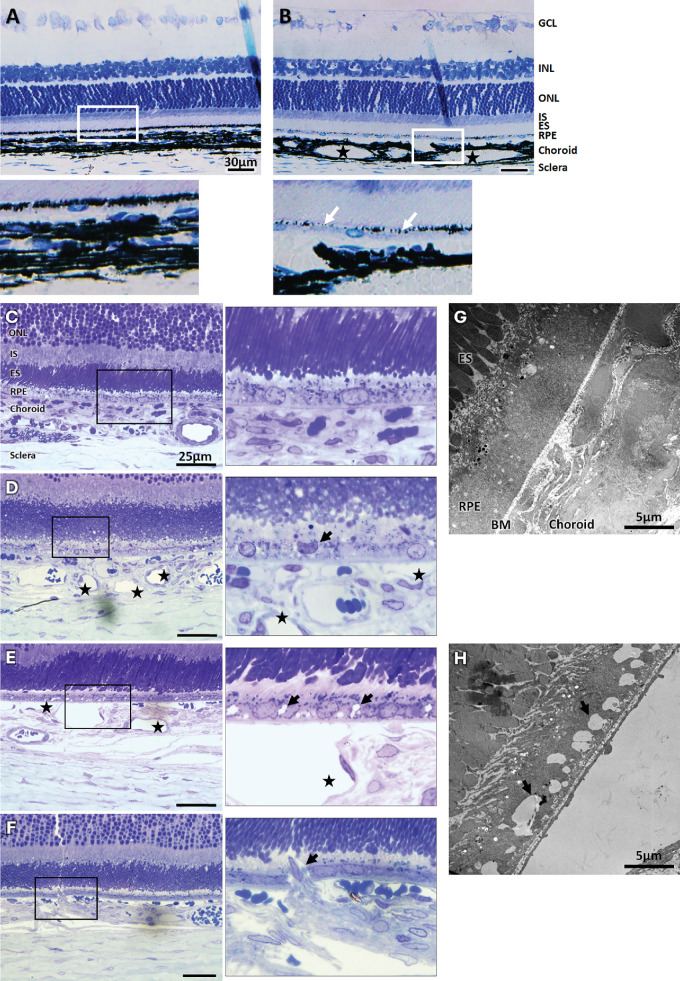
**Historesin and ultrastructural analysis of the retina-RPE-choroid complex in P1.hMR rats.** (**A**, **B**) Transversal posterior segment sections in historesin colored with toluidine blue. P1.hMR rats **B** show zones of vasodilation (*black stars*) and RPE melanosome loss (*white arrows*) compared to WT littermate animals **A**. Transversal semi-thin sections of eyes from WT littermates (**C**) or P1.hMR animals (**D**–**F**) colored with toluidine blue. Transgenic animals display important alteration of the RPE, including change in shape and size of RPE cells (**D** *inset, black arrow*), junctions opening (**E** *inset, black arrow*), and local RPE rupture (**F** *inset, black arrow*). Important choroidal vasodilatation can be observed (**D**, **E**, *black stars*) as well as abnormalities in the external photoreceptor segments orientation **D**. TEM analysis in WT (**G**) and P1.hMR rats (**H**) are consistent with semi-thin observations, where vacuoles are noticeable within RPE cells (*black arrow*) **H** with important choroidal vasodilation just below. GCL, ganglion cells layer; INL, inner nuclear layer; ONL, outer nuclear layer; IS, internal segment of photoreceptors; ES, external segment of photoreceptors.

Historesin, semi-thin, and TEM studies expose significant alterations in the retina-RPE-choroid complex of P1.hMR rats compared to WT littermates (see Fig. 8). Anatomic analysis of historesin sections show that P1.hMR rats display an important choroidal vasodilation and a loss of RPE apical melanosome compared to WT littermate animals (see Figs. 8A, 8B). Semi-thin sections show changes in RPE cell shape and size, with junction opening and local RPE rupture in P1.hMR animals (see Figs. 8B–D). TEM ultrastructural analysis confirms the presence of vacuoles/opened junction within RPE and prominent choroidal vasodilation at the level of the choriocapillaris in P1.hMR rats (see Fig. 8F). Abnormalities in external photoreceptor segments are observed (see Fig. 8B), indicating the presence of undigested segment and potential RPE disfunction. In rare cases, we could observe an unexpected finding with cells, originating from the choroid, most probably corresponding to Schwann cells, that are oriented toward the RPE, creating a disruption of the RPE barrier, as if a choroidal neoinnervation was growing from the choroid toward the outer retina (see Fig. 8D and inset).

**Figure 9.**
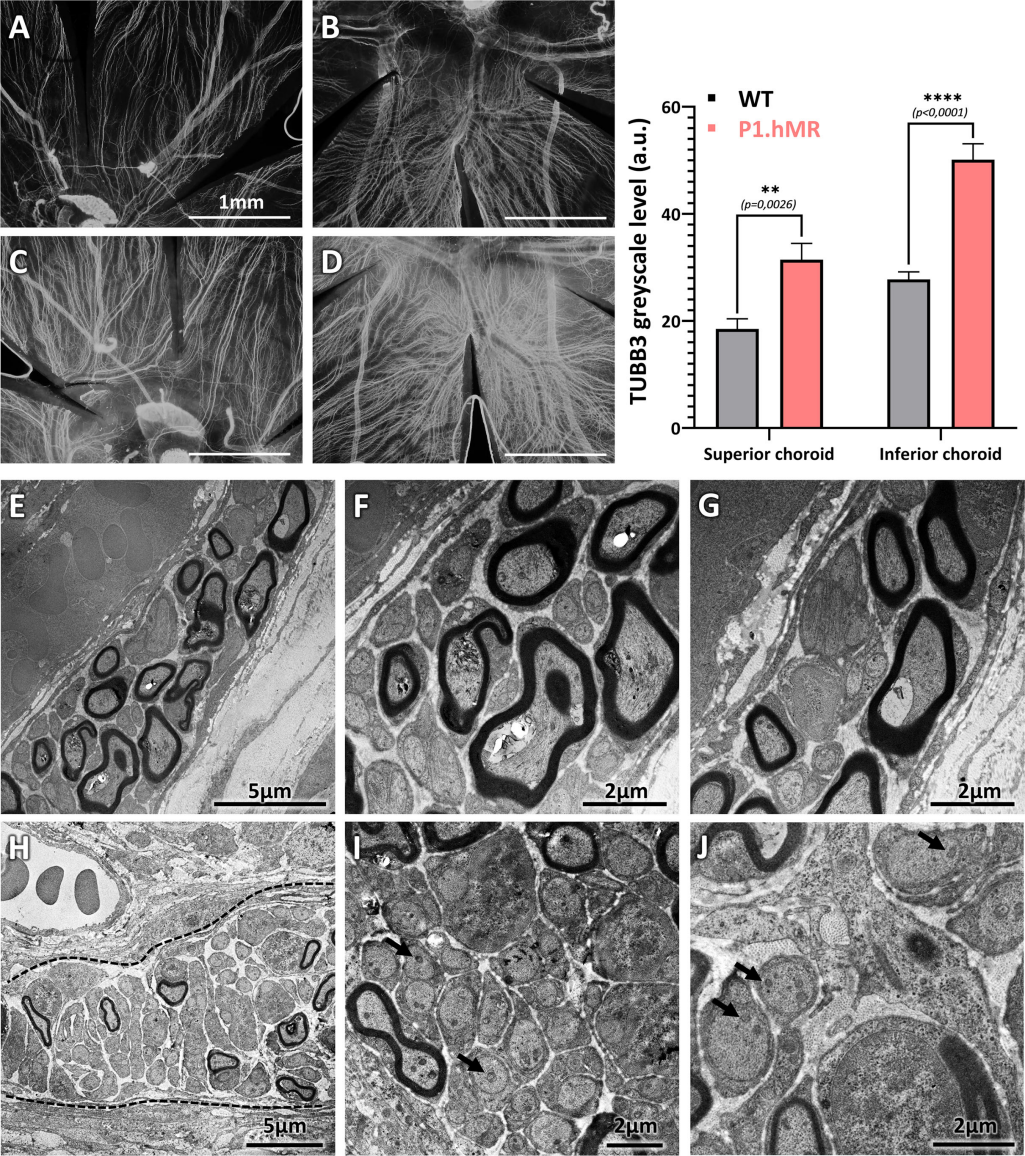
**Choroidal nerve density and choroidal neuropathy signs in P1.hMR rats.** (**A**–**D**) Choroidal nerve density was evaluated following TUBB3 immunostaining and greyscale level measurements in superior and inferior choroid. P1.hMR animals **C** and **D** show a significant increase (Graphics) in nerve density compared to WT littermate animals **A** and **B**. (**E**–**J**) Choroidal nerve ultrastructure was observed in TEM. The P1.hMR animals (**H**–**J**) display a noticeable reduction in myelinated fibers in large nerve (*black dotted line*, **H**) compared to WT littermates **E**. In addition, mitochondrias appear bigger and darker in P1.hMR rats (**I**, **J**, *black arrows*) compared to WT littermates (**F**, **G**). These observations could indicate a deregulation and a neuropathy within the choroidal innervation under hMR overexpression.

To further explore the choroid of P1.hMR rats, TUBB3 immunostaining was performed on whole-mounted choroid. This staining is targeting neuronal microtubules and allows to visualize the entire choroidal innervation (Fig. 9). Greyscale level quantification in the superior and inferior choroid demonstrate an increase innervation in P1.hMR rats (see Figs. 9C, 9D) compared to WT littermates (see Figs. 9A, 9B). TEM studies were conducted on transversal eye sections to evaluate large choroidal nerve ultrastructure. In the rare large nerves found, P1.hMR animals exhibit a noticeable reduction in myelinated fibers in large nerve (see Fig. 9H) compared to WT littermates (see Fig. 9E). In addition, the axonal mitochondria appears bigger and darker in P1.hMR rat (see Figs. 9I, 9J, black arrows) compared to WT littermates (see Figs. 9F, 9G), which is considered as a classical marker for neuropathy.

## Discussion

We and others have shown that aldosterone is pathogenic for the neural retina. At a low dose, aldosterone mostly regulates genes involved in ion and water homeostasis in retina macroglia causing edema, whereas at a higher dose, it causes vascular leakage and activates inflammatory cells within the retina.^10^^,^^14^^,^^28^^,^^34^ However, whether aldosterone could reach the neural retina at such high concentrations, even in pathologic conditions, is uncertain and whether MR activation by cortisol can produce similar inflammation in the retina than aldosterone was unknown. In this study, we found that contrarily to what could be expected, MR overexpression and aldosterone excess result in different transcriptional regulations and in different retinal phenotypes.

### Overexpression of MR in P1.hMR Rats Causes Low-Grade Retinal Inflammation and RPE/Choroid Pathology Mimicking Human Pachychoroid

Transgenic rats (on pigmented and albinos backgrounds) overexpressing the hMR (P1.hMR rats) have been generated to study the consequences of MR/GR balance dysregulation on the posterior segment of the eye under exposure to endogenous corticoid ligands. Corticosterone, measured in the rat ocular media^15^ and aldosterone, possibly present at undetectable level, could both activate MR in the neural retina,^35^ whereas choroid is exposed to higher levels of circulating gluco- and mineralocorticoids. In the transgenic rat eye, the highest hMR expression was measured in the neural retina and in the cornea with a lower expression level in the choroid/RPE complex. Despite this high expression level in the neural retina, no clinical signs of retinal inflammation, no retinal vascular leakage, and no alteration of the ERG response were detected, at least until 1 year of age, although signs of low-grade retinal inflammation were significant in the neural retina. This demonstrates that cortisol can induce inflammation but at a much lower intensity than aldosterone. A proinflammatory microglia phenotype was observed together with upregulation of microglia-related genes, such as the dual specificity tyrosine-phosphorylation-regulated kinases (*Dyrk1A*) that contributes to inflammation through TLR4/NF-κB P65 pathway^36^ or the downregulation of versican that could be proinflammatory through the TLR2-mediated NF-κB pathway activation.^37^ On the other hand, microglia activation seemed counter balanced by other regulations, such as the downregulation of Spp1 that encodes osteopontin.^38^ The retinal inflammation phenotype seemed more pronounced in female rats, where activated microglia were masked by a substantial amount of distinct IBA1+ cells. These cells could correspond to infiltrated macrophages, consistent with the transcriptomic analysis and the upregulation of the *Herpud1* gene discussed below.

Although hMR expression level was lower in the RPE/choroid, pathologic clinical changes were detected close to those described in the uninephrectomy-aldosterone-salt mouse model^26^ and in the P1.hMR mouse model,^17^ suggesting that aldosterone contributes to the pathologic phenotype in this tissue. A radical modification of IBA1+ choroidal cells that could correspond to macrophages or to dendritic cells^39^ was notable. The choroidal vasodilation could result from a direct aldosterone effect on endothelial/vascular smooth muscle cells,^11^ from histamine released by the increased number of choroidal mast cells,^40^ from signals originating from the retina (discussed below), and from deregulated neural regulation because choroidal neuropathy is observed in the P1.hMR rat, similar to what was observed in the P1.hMR mouse model.^31^ Increases in choroidal nerve density, myelin sheets’ modification, and abnormal axonal mitochondria demonstrated further the neuropathogenic role of MR on autonomous nerves structures. Interestingly, intravitreal aldosterone injection negatively regulated pathways in axon guidance (KEGG), axonal transport of mitochondrion (GOBP), mitochondria fatty acid beta oxidation (Reactome), and positively regulates the pathway in mitochondrial calcium ion transport (Reactome) and transcriptional activation of mitochondrial biogenesis (Reactome) in the retina suggesting that MR could regulate mitochondria functions.

The RPE cells from P1.hMR rats showed abnormal ICG transport,^33^ which is in line with ICG leakage and ICG-labeled dots on ICG-A. In RPE cells, focal disorganization, change in their pigmentation, accumulation of lipofuscin, vacuole and junction openings, and elongation of undigested outer segments, is very similar to the phenotype of RPE cells in uninephrectomy-aldosterone-salt mouse model^26^ and in humans with pachychoroid pigment epitheliopathy^41^ who have a well preserved retina despite significant RPE/choroid alteration. In this study, we have not performed molecular analysis of the RPE/choroid as done recently in the P1.hMR mouse model,^31^ but genes encoding proteins that play major roles at the RPE/neural retina interface and regulating P1.hMR neural retina physiology, could contribute to the epitheliopathy. The chloride intracellular channel 4 (CLIC4), enriched at apical RPE microvilli and crucial to proper RPE/retina interface^42^ is upregulated in P1.hMR rats and by aldosterone. The glutathione specific gamma-glutamylcyclotransferase 1 (CHAC1) is found upregulated both in the aldosterone model and in P1.hMR rats, this protein plays a pivotal role in RPE cells ferroptosis with oxidative stress damages.^43^ The retbindin protein, expressed in the rods and present at the RPE/retina interface, is downregulated in P1.hMR rats, potentially reducing flavin levels in the neural retina, which could favor long-term retinal damages and premature retinal aging.^44^ In Rtbdn-knockout (KO) mice, retinal development and ERG were normal but with aging, ERG amplitudes declined, photoreceptor outer segments became disordered, RPE developed vacuoles, and lipid, protein, and calcium deposits were reminiscent, along with activation of microglia.^45^ Whether ERG changes will occur with aging and/or under light exposure in the P1.hMR model is under evaluation.

The RPE/choroid pathogenic changes despite lower levels of hMR expression could be related to the activation of MR by different ligands and more specifically to a higher exposure of MR to circulating aldosterone, although efflux proteins that limit aldosterone availability are present at the outer retinal barrier.^46^ The transcriptional regulations induced in RPE/choroid by aldosterone has been previously published^26^ and could occur in patients with high circulating aldosterone levels. Signals originating from the neural retina might also contribute to the epithelio-choroidopathy, as several genes regulated in the neural retina of P1.hMR rats but not by the aldosterone model encode proteins that intervene in the function of the RPE/choroid. *Herpud1*, among the most upregulated genes in the neural retina, is a protein coding gene involved in the unfolded protein response and in the destruction of misfolded proteins. *Herpud1*, upregulated in IL-4-treated macrophage, promoted their migration, and, in a mouse model of choroidal neovascularization (CNV), it promoted M2 macrophage polarization, favoring the development of CNV.^47^^,^^48^ Polymorphism in the *Herpud1* gene was associated with polypoidal choroidal vasculopathy and HERPUD1 was expressed in the subretinal vascular membranes.^49^

Another gene involved in pathological angiogenesis and choroidal dilation, which is upregulated in P1.hMR neural retina but not by aldosterone, is the endothelial Pas domain protein 1 (*Epas1*), which encodes a paralog of HIF-1a, involved in the post vasculogenesis stages.^50^ Patients with a gain of function mutation in *Epas* genes showed, among other signs, retinal pigment epithelium changes and thickening of the choroid with dilation of choroidal vessels,^51^ similar to what is observed in P1.hMR rats. *Ephx*2, highly upregulated in the P1.hMR rat retina (X7), is a proinflammatory enzyme that converts cytochrome P450-derived epoxides of fatty acids to the corresponding diols. It is upregulated in photoreceptors and RPE cells of patients with wet AMD and Ephx2 knockdown significantly reduced CNV and the expression inflammatory markers.^52^ Finally, high-temperature requirement A1 (*Htra1*), increased in the P1.hMR rat retina, encodes a secreted serine protease and has been identified as a disease-susceptibility gene for AMD, including polypoidal vasculopathy. Transgenic mice overexpressing HtrA1 showed increased macrophages, RPE cells’ migrations, and increased laser-induced neovessels,^53^ whereas transgenic mice overexpressing human HTRA1 became more susceptible to RPE damage caused by oxidative stress through damaging mitochondrial function and activating HIF-1 signaling.^54^ Altogether, these regulations tend to show that signals originating from the neural retina of P1.hMR rats could intervene in the pathologic phenotype observed at the level of RPE/choroid.

### Aldosterone Versus hMR Overexpression of Common and Divergent Transcriptomic Signatures

Although both aldosterone and cortisol bind to MR and activate MR, aldosterone excess or MR activation by endogenous cortisol result in different genes regulations. Acute intravitreal aldosterone injection and hMR overexpression, respectively, regulated 1947 and 344 genes within the retina, with only 35 common genes. Among these 35 common genes, 14 are upregulated and 21 are downregulated in P1.hMR animals, whereas the majority are upregulated in the aldosterone model (29 genes) with only 6 genes are downregulated. These results highlight important differences that probably depend not only on the acute/chronic aspect of the models, but also greatly rely on the ligands/receptors (MR and GR) and co-factors’ interactions, that is based on binding affinity but also on interaction kinetics and on possible heterodimers formation.^55^ Injection of aldosterone positively regulates pathways, such as response to mineralocorticoid (GOBP) and response to aldosterone (GOBP), but also the cellular response to GCs stimulus. The positive regulation of the GR pathway could be explained by an increase of GR pathway activation, in response to aldosterone-induced retinal inflammation. Overall, the intravitreal aldosterone injection model is mostly representative of an aldosterone-on-MR transcriptomic signature, whereas hMR overexpression is mostly representative of endogenous GCs-on-MR impact in the retina and therefore closer to clinical phenotypes when no major disruption of retinal barriers disrupts the ocular steroidome.

Only in the aldosterone injected condition, genes related to proinflammatory processes are regulated, such as the IL-6 and IL-6-signaling protein STAT3^56^ and CXCL2 and CXCL3.^24^ In addition, S100A9, the neuronal pentraxin Nptx2, Pentraxin-3, and Galactin-3, which both interact with the recognition receptor TLR4, are all upregulated and known to play a role in microglia inflammatory activation.^57^^,^^58^ Upregulation of Nos2, arg1, CD86, and TSPO (translocator protein) indicate a shift toward proinflammatory M2-type retinal microglia,^59^^,^^60^ consistent with positive regulation of the IL-6-JAK-STAT3 signaling (HALLMARK) and regulation of microglial cell activation (GOBP).

But proinflammatory genes were also upregulated in both the aldosterone model and in the P1.hMR neural retina, such as the ATF3, that acts as a transcription factor involved in cellular stress response linking inflammation, oxidative stress, and immune responses^61^^,^^62^ and identified as a central mediator of the retina-resident mononuclear phagocytes reprogramming following peripheral inflammation. ATF3 polarized the phagocytes toward a proangiogenic phenotype that aggravated CNV in the aging retina.^63^ *Trib3*, which is also upregulated in the two models, plays a major role in the blood-brain barrier breakdown.^64^ Interestingly, *Trib3* contributes to the survival and activity of mast cells,^65^ which number is increased in P1.hMR rats, whereas pathways related to mast cell activation (GOBP) are positively regulated in an aldosterone model. On the other hand, there were inflammatory genes regulated in the opposite way in the two models. Particularly *Spp1*, encoding osteopontin, that is a known aldosterone and MR-induced gene in numerous tissues, including the kidneys^38^ and the retina,^13^ was upregulated by aldosterone but downregulated in the P1.hMR rat retina. Osteopontin was upregulated in retinal microglia and macrophages in neovascularization models,^66^ elevated in the macula with late dry and wet AMD, and correlated with inflammatory pathways in the retina.^67^ Furthermore, reduction of SOCS3 increased SPP1 expressing macrophages and microglia.^66^ In the diabetic rat model, local MR antagonist reduced *Spp1* expression,^13^ showing that it is an MR target gene. In the retina of P1.hMR, the downregulation of *Spp1* could be one of the mechanisms that limits inflammation. Interestingly, osteopontin could be used as a biomarker to differentiate GCs with high or low MR activation potential in the retina, which should be further studied.

Genes only upregulated in P1.hMR rats retinas include *Ddit4/Redd1* involved in responses to cellular stress, including hypoxia and DNA damage. In the diabetic retina, REDD1 promoted NFkB signaling and inflammation an increased NLRP3 activation in Müller cells.^68^^,^^69^ REDD1 has been identified as one of the major drivers of GCs-induced skin atrophy^70^^,^^71^ and, interestingly, we showed that MR antagonists efficiently promoted the healing of GCs-induced skin ulcers,^72^ potentially linking REDD1 with the MR pathway activation in the skin. The dual specificity tyrosine-phosphorylation-regulated kinases (DYRKs) 1A (*Dyrk1A*), also was upregulated in the P1.hMR rat retinas, was shown to contribute to LPS-induced brain neuroinflammation via the TLR4/NF-κB P65 pathway, microglia activation, and neuronal damage.^36^

We recognize several limitations in this study. Cautious comparison should be done as aldosterone injection is an acute event and the transgenic P1.hMR model results in a chronic overexpression of the human MR, under the P1 promoter, which favors but does not restrict its expression in the brain and in the retina. Whether MR overexpression in the brain modifies the ocular steroidome of the rat is under investigation. Aldosterone acute intraocular injection was used as an in vivo model to identify the specific effect of aldosterone on MR. This can represent an inflammatory mechanism in cases of blood-retinal barrier disruption, that could favor aldosterone entry in the retina. As aldosterone is not the main MR ligand, the P1.hMR rat model was used to mimic a more clinically relevant phenotype. Indeed, MR overexpression was shown in human eyes with AMD and diabetic retinopathy, whereas increased cortisol levels were measured in diabetic ocular media, suggesting that MR pathway overactivation by cortisol occurs in humans.^13^^,^^22^ In this multifactorial diseases, low grade inflammation was described and is similar to the one we are describing in the animal model. Another limitation is the lack of measurements of the levels of Corticoids and sexual hormones in the animal models. It cannot be excluded that in P1.hMR rats, the production of corticoid hormones and progesterone could be modified,^73^ which could explain the sexual dimorphism that should be further explored in depth.

Our experimental design did not include the intraocular injection of aldosterone or GCs as we did show that, in P1.hMR mice, the long-lasting GCs triamcinolone, did aggravate the phenotype.^31^ In further studies, we plan to phenotype old P1.hMR rats and to measure their ocular steroidome before performing additional steroid challenges.

In conclusion, in the neural retina, aldosterone excess and MR overexpression regulate differential proinflammatory pathways. A more pronounced inflammation is observed when aldosterone activates MR, whereas a low-grade neuroinflammation is occurring when MR is overactivated by endogenous ligands. The effects of MR pathway overactivation are more pronounced in the RPE/choroid complex, that is exposed to aldosterone, compared to the neural retina. This could be explained by the fact that GCs mostly activated MR in the neural retina whether both aldosterone and cortisol activate MR in the choroid. Understanding the pro versus anti-inflammatory effects of corticoid receptors in the retina could help design optimized drugs, whereas the specific transcriptomic MR-targets could be used to select GCs with high GR and low MR activity.

## Supplementary Material

Supplement 1

Supplement 2

Supplement 3
