# Supplementary material for: Differential Effect of Aldosterone or Mineralocorticoid Receptor Overexpression on Retinal Inflammation
Source: Invest Ophthalmol Vis Sci. 2024 Oct 25;65(12):39. doi: 10.1167/iovs.65.12.39 (PMC11512573; doi:10.1167/iovs.65.12.39)
Supplement: Supplement 1 [file iovs-65-12-39_s001.pdf]

Supplementary online information for

Differential effect of aldosterone or mineralocorticoid receptor overexpression on retinal inflammation

Bastien Leclercq[1], Dan Mejlachowicz[1], Linxin Zhu[1], Laurent Jonet[1], Chadi Mehanna[3], Marianne Berdugo[1], Theano Irinopoulou[4], Fréderic Jaisser[5], Min Zhao[1], Francine Behar-Cohen[1, 2]

[1]Centre de Recherche des Cordeliers, INSERM UMRS1138, Université Paris Cité, Sorbonne Université, team « Physiopathology of ocular diseases : Therapeutic innovations », Paris, France.

[2]Ophthalmopole Cochin University Hospital, Assistance Publique-Hôpitaux de Paris, France.

[3]Hôpital Américain de Paris, 55 Boulevard du Château, 92200 Neuilly-sur-Seine, France.

[4]INSERM UMR-S 1270, F-75005 Paris, France; Sorbonne University, 75005 Paris, France; Institut du Fer à Moulin, 75005 Paris, France.

[5] Centre de Recherche des Cordeliers, Inserm, Université Paris Cité, Sorbonne Université, team « Metabolic Diseases, Diabetes and co-morbidities », Paris, France.

Corresponding author

Francine Behar-Cohen, 15 rue de l'Ecole de Médecine 75006 Paris, France, francine.behar@gmail.com

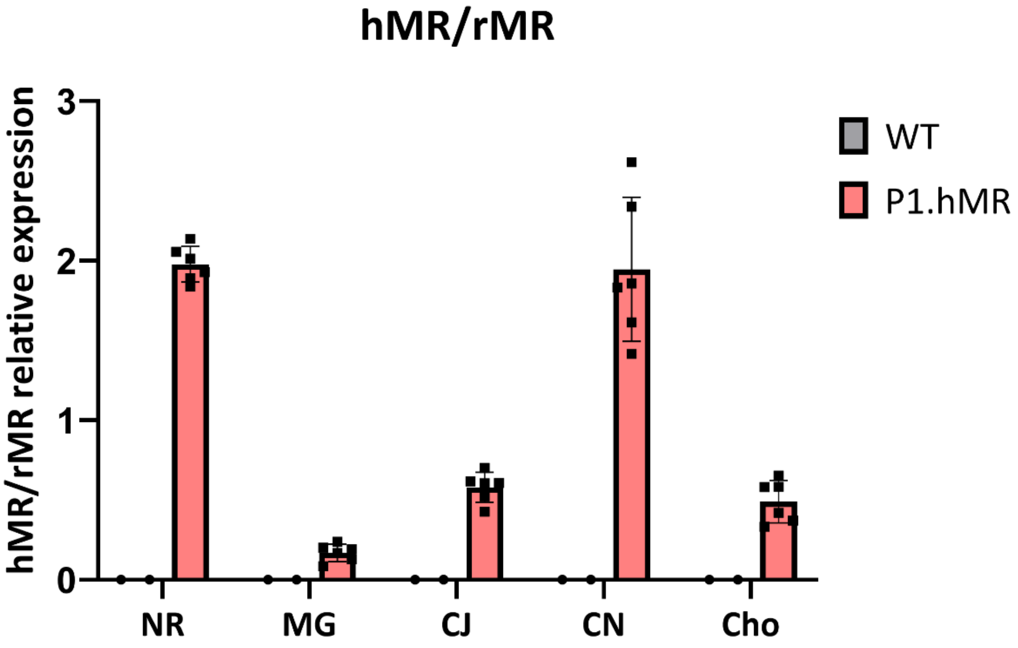

Supplementary figure 1 : qPCR quantification of hMR transgene in various ocular tissues. RNA from different ocular tissues were isolated using the RNeasy Mini Kit (Cat. Nb. 74106, QIAGEN), followed by DNase I treatment (Cat. Nb. 79254, QIAGEN) according to the manufacturer's protocol. First-strand complementary DNA was synthesized from the total mRNA utilizing random primers (ThermoFisher Scientific) and SuperScript II reverse transcriptase (ThermoFisher Scientific). Transcript levels were then assessed through quantitative real-time PCR conducted on the QuantStudio™ 5 Real-Time PCR system (Applied Biosystems, Foster City, CA, USA) with SYBR Green detection. Relative quantification of results was achieved through the Delta CT threshold calculation method. We analyzed the expression of transgene human *Nr3c2* (encoding hMR) and rat gene *Nr3c2* (encoding rMR), across different ocular tissues, namely the neural retina (NR), the meibomian gland (MG), the conjunctiva (CJ), the cornea (CN) and the choroid/RPE complex (Cho). Reference genes Hprt1, Ubc, and 18S were utilized for normalization.

List of primers used in this study

Gene Name	Forward/Reverse	Sequences
18S	Forward	TGCAATTATTCCCCATGAACG
18S	Reverse	GCTTATGACCCGCACTTACTGG
Ubc	Forward	ATCTAGAAAGAGCCCTTCTTGTGC
Ubc	Reverse	ACACCTCCCCATCAAACCC
Hprt1	Forward	GCGAAAGTGGAAAAGCCAAGT
Hprt1	Reverse	GCCACATCAACAGGACTCTTGTAG
Nr3c2	Forward	TAAGTTTCCCCACGTGGTTC
Nr3c2	Reverse	ATCCACGTCTCATGGCTTTC

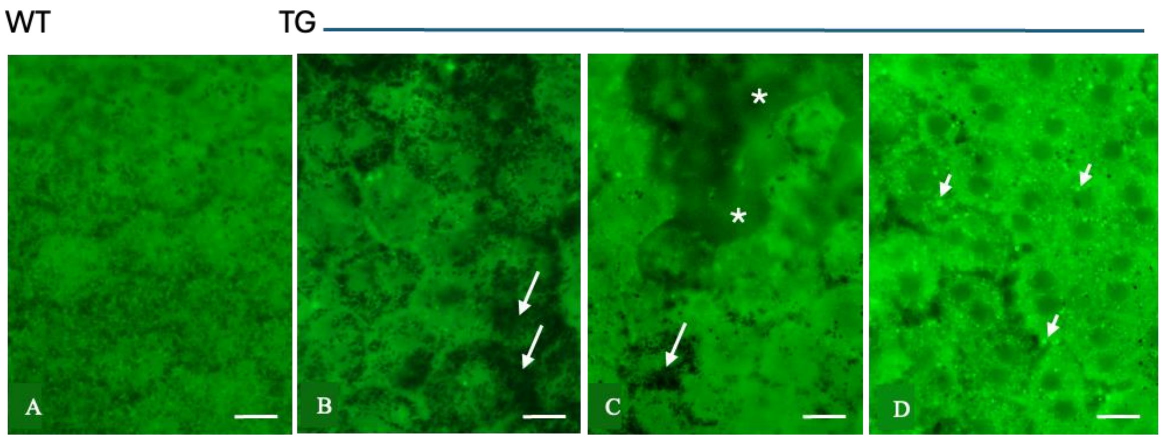

Supplementary Figure 2: Autofluorescence of flat-mounted RPE from WT and P1.hMR rats. WT pigmented rats show homogenous hyperautofluorescence (green) with homogenous distribution of melanosomes (black) (A). In P1.hMR pigmented rats (TG), there were areas of agglomerated pigments (B, C white arrows), areas of missing pigments and disorganized cells (C, stars) and areas with intense hyper autofluorescence due to loss of pigments and accumulation of lipofuscin granules (D, white arrows). Scale bar: 25µm.

Supplementary file 1: Processed transcriptomic data obtained from bulk RNAseq of neuroretinas after injection of aldosterone or saline (sham).

Supplementary file 2: Raw and processed transcriptomic data obtained from bulk RNAseq of P1.hMR and WT littermates neuroretinas. The data discussed in this publication have been deposited in NCBI's Gene Expression Omnibus and are accessible through GEO Series accession number GSE266379 (https://www.ncbi.nlm.nih.gov/geo/query/acc.cgi?acc=GSE266379)
